# The Modern Outlook on Mental Health
*An address given at Bristol University on Thursday, 12th March, 1931, to a meeting under the auspices of the National Council of Women.


**Published:** 1931

**Authors:** L. G. Brock

**Affiliations:** Chairman of the Board of Control


### THE MODERN OUTLOOK ON MENTAL HEALTH.*

BY

L. G. Brock, C.B.,

*Chairman of the Board of Control.*

---

There are few questions of public health which are more in need of frank discussion than the question of mental health. How important this question is you will realize when I tell you that there are more than 120,000 patients under care in our mental hospitals in England and Wales, and it is estimated that in addition there are 300,000 mental defectives, of whom roughly one-third need institutional care.

At the outset I want to remind you of the distinction between mental disease and mental deficiency. This distinction has long been recognized in English law, and as far back as the reign of Edward I we find the statute (*De praerogativa regis*) enacting that "The King shall have the custody of the lands of natural fools." The same statute enacts that :—

> "The King shall provide when any that beforetime hath had his wit and memory happen to fail of his wit, as there are many that have lucid intervals, that their lands and tenements shall be safely kept without waste and destruction."

* An address given at Bristol University on Thursday, 12th March, 1931, to a meeting under the auspices of the National Council of Women.

The difference between the mental defective, the natural fool, and the person suffering from mental disorder could not be better put. Perhaps I ought to apologize for having, of necessity, to say much that will be familiar to many who are present here this evening. For the sake of clearness and completeness I am bound to emphasize what to some of you must be elementary distinctions. But these distinctions are, after all, fundamental, because they affect the kind of provision which the community has to make. Speaking broadly, mental disease calls for treatment and mental deficiency for custodial care and training. Disease may be remedied; defect, at any rate in the present state of our knowledge, is permanent.

Dealing first with mental disease, the main statute is the Lunacy Act of 1890. That Act had three main objects: to protect the community from persons unfit to be at large; to protect the patient from maltreatment and improper detention; and to protect the patient's property. The test of liability to detention is behaviour. The point is important because there is a good deal of misunderstanding about it. Nobody is detained in a mental hospital because of his opinions, however misguided they may be. Political life would indeed be dangerous if it were otherwise. Delusions do not matter until they affect the patient's conduct. The crucial question is whether the patient is dangerous to himself or to others. I want to emphasize, too, that the operative document is the reception order which is made by a judicial authority. There are medical certificates, but the effective instrument is the document signed, not by a doctor, but by a magistrate. The Act of 1890 was primarily concerned with custodial care.

It does, indeed, recognize the possibility that the patient may recover, but it takes little account of remedial treatment. You can read the Act from beginning to end—and it is a very long one—without realizing that it deals with sick people in need of medical treatment.

The Act of 1890 remained in force without substantial amendment for forty years, until the passing of the Mental Treatment Act last year. That Act made several most important changes. First of all, it empowered local authorities to provide out-patient clinics. This marks the beginning of an effort to treat mental disorder on preventive lines. Secondly, it provides for the admission of voluntary patients to public mental hospitals, and so gives everybody, whatever his means, a privilege which hitherto had been the monopoly of the well-to-do. In the third place, it recognizes an entirely new class of patient, the temporary patient dealt with under Section 5. This Section provides for treatment in certain cases solely on medical recommendation and without the intervention of any judicial authority. It is, in fact, the first attempt to assimilate the treatment of physical and mental disorders. It is a statutory recognition of the fact that mental disease is like physical disease, a condition which calls for medical care, which may be and ought to be treated, the necessity for treatment being determined by doctors and not by lawyers. Treatment as a temporary patient is limited to non-volitional cases, that is to say, patients who are unable either to consent or to refuse. The practical effect of this provision is to obviate the need for certification in many confusional, toxic and puerperal cases of relatively short duration. The woman who has a short period of mental disturbance after child-birth or the man who breaks down for a time after influenza can in the future escape the stigma of certification.

It is most important that medical men should recognize the new opportunities which the Act gives them. Parliament in the past has sometimes been distrustful of doctors, but in the Mental Treatment Act it trusts them as they have never been trusted before in our lifetime. I hope they will justify this confidence, and so pave the way for the complete disappearance of the judicial authority so far as early cases are concerned. Doctors, if I may be allowed to say so, are timid of responsibility, and I admit that recent cases in the courts have afforded some justification for this. But we have tried to meet their difficulties by giving them additional protection against vexatious and unwarrantable legal proceedings, and I hope the general practitioners will face their responsibilities, and will not try to pass them on to others.

Now I come to the place of the voluntary and municipal hospital in the treatment of mental disorder. One of the biggest changes made by the Mental Treatment Act is to associate these hospitals in the treatment of mental disorder. They can co-operate in three ways: by providing out-patient clinics; by admitting voluntary patients; and by admitting temporary patients. The out-patient clinic is simple, especially if the social work can be done by voluntary organizations. All that is needed is a consulting-room and a share of a waiting-room. There are many advantages in treating a clinic as part of the general out-patient department. Patients can go there without exciting curiosity. It is most important that they should be free to seek advice without provoking embarrassing questions from their neighbours. Many other patients will be referred to the clinic by the medical staff of the hospital. I want to see the senior staffs of the mental hospitals co-operating in these clinics, and they should be appointed on the honorary staff of the voluntary hospital. In return, the staff of the voluntary hospital should become consultants at the mental hospital. I am convinced that this closer association will be good both for the doctors and the patients, particularly as many of the patients are physically as well as mentally sick. But the out-patient clinic will soon reveal cases which the physician will want to keep for a more complete examination. I doubt if any clinic can be regarded as really complete unless it is supplemented by a few observation beds. This has been the experience in Oxford and in a few other centres where out-patient clinics were opened before the passing of the Mental Treatment Act. Observation beds are valuable, but by themselves they are not sufficient. We need, in particular, to provide for the admission as voluntary patients of those persons whose mental disorder is complicated by physical conditions which require more resources in men and equipment than a mental hospital may be expected to provide.

So far I have spoken of voluntary patients, many of whom could be nursed in an ordinary ward by general-trained nurses. But there will be cases, particularly toxic cases, who will be too confused to be able to consent to treatment, and can therefore only come in as temporary patients. These will need nurses specially trained in mental nursing, and the number of hospitals able to provide for them will necessarily be limited. But it seems desirable that teaching hospitals, at any rate, should make some provision for temporary patients, for the sake of the student. The great majority of medical students will go into general practice, and every doctor in general practice has, sooner or later, to deal with incipient mental disorder. It is vitally important that he should learn to recognize it early, yet it is admitted that at present the opportunities of clinical study are lamentably inadequate. For this reason I am convinced that mental wards would have special value in teaching hospitals.

But closer association between psychological and general medicine has other advantages besides its value from the point of view of medical education. Psychological medicine has far too long been kept, as it were, in a watertight compartment, and anything which helps to break down the barrier between psychological and general medicine is a clear gain. In particular, we need more study of the relation between physical and mental disorders, and there can be little doubt but that research in this direction would throw light on the causation of mental disorders.

All this may have suggested to you that under the new Act the treatment of early cases is to be concentrated in the voluntary hospitals. This is far from being the case. The majority of cases will continue, and rightly continue, to be treated in public mental hospitals. But if they are to have a fair chance, our public mental hospitals must be properly equipped for active treatment on modern scientific lines. To this end it is most important that every mental hospital should have a separate admission unit and treatment centre, supplemented by convalescent villas. Now how does Bristol stand ?

You are in a position of special difficulty. Your present hospital at Fishponds is, for the most part, an old building, and the amount of land available is so restricted that it is practically impossible to modernize it. The City Council, recognizing this, have secured a site for a new institution at Barrow Gurney. This is a development of the greatest value, because it will give an opportunity of providing the treatment unit and the therapeutic resources which you cannot at present provide at Fishponds. But it will be essential to maintain close relations between the two institutions, and to make them, as it were, dovetail into one another.

If these ideals are to be realized you will need to educate public opinion. Even the most progressive local authorities need the support of enlightened public opinion, and an organization such as the National Council of Women can do a great deal to bring home to people the need for organizing the treatment of mental disorder on progressive lines. Of course, I know that these new developments mean expense, but I am convinced that in the long run they mean economy. After all, nothing is more expensive than to turn a curable case into a hopeless chronic whom you will have to maintain for the rest of his life—which may be, and often is, a very long one. There are some kinds of economy which are positively criminal.

People often ask, What is the good of all this expenditure ? They admit that patients recover, but they ask whether there are really any cures. I agree that some patients recover in spite of their doctor. That is true in all branches of medicine. But it is sheer blank fatalism to argue that because there are unexplained recoveries, and often unaccountable failures, that there is no need for modern therapeutic equipment. If you ask whether there are any cures, I would instance the treatment of general paralysis of the insane. That is a disease which accounts for about 10 per cent. of male admissions and 6 per cent. of female admissions into our mental hospitals. Up till a few years ago, though there were sometimes temporary remissions, general paralysis of the insane meant death in the course of two, three or four years. Now, thanks to the treatment by induced malaria, there are many men and women back at work and leading useful lives who but for this would long ago have died miserably. I do not say that there is a complete cure. No one can repair damage already done, any more than you can repair a damaged heart. But what you can do is to arrest this disease and, in many instances, send the patient back into the world.

Even if I were doubtful about the therapeutic value of this treatment or that, I should still feel justified in trying it for the sake of the psychological effect on the patient. Nothing is quite so fatal as to make the patient or his friends feel that nothing can be done. There is no use in medicine for the pessimist. The doctor who does not believe in himself will never make his patients believe in him, and the doctor who feels that he can do the patient no good generally turns out to be a true prophet. I have had twenty years' close association with doctors, and I still believe, more than I ever did, in the possibilities of medicine. By "medicine" I do not mean drugs, I mean the whole art and science of medicine. Miracles do happen; I have seen them. But they only happen to the people who deserve them and work for them. The recovery rate is not as good as it ought to be; it is not as good as it is going to be. It can be improved, and it must be improved. But the average recovery rate of 30 per cent. of admissions compares favourably with the results in the medical wards of general hospitals. We are apt to forget how the recovery rate in general hospitals is inflated by the inclusion of large numbers of comparatively straightforward surgical cases. After all, the chance of recovery from mental disorders is far better than the chance of recovery from heart disease, cancer or disseminated sclerosis.

Now I turn to the other side of the picture—mental defect. Here we are up against a really appalling social problem. According to the investigation carried out by Dr. Lewis there are 300,000 defectives in England and Wales, of whom at least a third ought to be receiving institutional care. People sometimes ask me why we hear so much about mental deficiency now while so little was heard of it a generation ago. The real answer is that mental defect is not a new problem. What is new is the diagnosis, or rather the recognition of the need for making some provision for this most unfortunate class. It was the Royal Commission of 1904 which first focused attention on the fact that much destitution and not a little crime was due to subnormal mentality. It was the report of this Commission which led to the passing of the Mental Deficiency Act of 1913. The low-grade defective had long been recognized. You cannot overlook the idiot and the imbecile. What had not been recognized was the existence of a much larger mass of people who are the victims of arrested mental development and differing from inbeciles only in degree. Physiologically all defectives have the common feature that the brain is not completely developed. The condition is often inherited, or at any rate innate, but it may be due to injury at birth or in early life or to disease such as encephalitis lethargica. Psychologists tell us that the last part of the brain to develop is that which controls conduct. Hence defectives, irrespective of age, behave like children. They are impulsive, unstable, incapable of sustained effort or self-control. They may be cruel, vicious or erotic. But it is unfair to use language implying moral judgments. Defectives are not responsible for their actions, and they are amoral or, if you prefer the term, unmoral rather than immoral.

Modern social organization is very complex, and it demands a degree of self-control of which the defective is altogether incapable. In a sense, the higher grade defectives may be said to be the victims of civilization. In a more rudimentary society they would rub along without great difficulty, but they cannot submit to the restrictions which are inevitable in a more complex society. The real test of defect is social adaptability. I want to stress that point, because there is a very general, and entirely wrong, impression that people are classed as mental defectives and often segregated because they cannot reach some prescribed educational standard. What are called "intelligence tests" are used, and are, indeed, most valuable for the purpose of measuring the degree of defect. But educational failure is not conclusive evidence. No one is segregated for failing to pass some educational standard; and some defectives, particularly the moral defective, may be up to or even a little above the average intelligence as measured by intelligence tests. The real test is whether people can adapt themselves to social conditions.

"Social adaptability" is a very useful abstract phrase, but, like all abstract phrases in general use, it tends to become a kind of "shorthand," and to be used without any very definite meaning being attached to it. What does it mean in actual practice as applied to the individual case? I think the best answer is to ask another question. Where would the defectives be if no provision were made for them? Some would be in prison, often for silly, purposeless crimes. Rick-firing, for example, is four times out of five the work of defectives. It is very typical of the sort of thing which defectives do. Window slashing is another instance. You may remember that some time ago there was an epidemic of window smashing in London and elsewhere. I speak without authority, but my recollection is that, judging from the press reports, almost every one of the persons convicted of these offences exhibited some signs of mental defect. Then there will be many cases of sexual offences. I do not mean that all sexual offences are due to defect, but a great many certainly are. Many other defectives will be in workhouses. They form the great majority of the chronic unemployable. Many of the women will be prostitutes, drifting from the brothel to the workhouse and from the workhouse back to the brothel. Many will be spreading dirt and disease through sheer incapability of observing even the elementary laws of hygiene.

Now the low-grade defectives, idiots and imbeciles, occur at all social levels, but the high-grade defectives are mainly the product of 10 per cent. of the population. In my younger days we used to hear a great deal about the problem of "the submerged tenth." I think we are coming now to recognize that the real problem is the problem of the *subnormal* tenth, and it is more and more evident that the slum is to a large extent the creation of the slum mind and not merely the automatic result of a bad environment.

The National Council of Women is a practical body. You want to get things done, and you will naturally ask: What can be done for the defectives? About two-thirds can be dealt with by supervision or guardianship, but, as I have said, the remaining one-third require some institutional provision. The best way of providing for the young and trainable case is unquestionably the colony. Adapted Poor Law buildings will provide adequately for the older and unemployable cases. The great value of the colony is that it gives the defective an opportunity of enjoying community life. Many of them can be partly self-supporting. A well-organized colony will not only do its own washing, cooking and housework; it will do its own carpentry, bootmaking, dressmaking, weaving and market gardening. When I am asked whether, in these hard times, we can afford colonies, I am tempted to rejoin by asking whether we can afford to leave the defectives at large. The total cost to the community of mental defect cannot be calculated, because it cannot be separated from the general cost of maintaining prisons, hospitals and workhouses, but if you could calculate the cost separately the figure would be truly appalling. And so, if you ask whether we can afford colonies, I would say: "Can we afford to do without them?" Quite apart from the merely economic aspect of the question, if society, for its own convenience, restricts the liberty of some of its members, is there not a social obligation to make life tolerable for those who are segregated not merely for their own protection but also for the convenience of others?

What is Bristol doing in this matter ? You have provided, and will soon be opening, a very fine new colony at Hortham ; but you are also, I am sorry to add, accommodating many of your defectives, including children, at Stapleton, where I have seen quite young children being housed and taught in buildings which were erected for the accommodation of French prisoners in the Napoleonic Wars. But in addition to provision made by the Council, Bristol has an honourable place in the treatment of mental defect because of the pioneer work carried out at Stoke Park Colony.

What can the National Council of Women do for mental defect ? There is one special sphere of work open to women, and that is social service, both in relation to the out-patient clinic and to after care. We need a nucleus of highly-trained women for this purpose, but there is also room for the voluntary worker, though the voluntary worker is also none the worse for training if she can afford to take it. It is work which requires tact and patience, but it is vital to the success both of the out-patient and after care departments. Much the same type of worker is needed to assist in the ascertainment and supervision of those mental defectives who do not require institutional care. I know there is a tendency to leave all work of this kind to paid experts, but I should be sorry if this became general. The voluntary worker may not be so highly trained, but the voluntary spirit is worth preserving. The Mental Treatment Act gives local authorities power to contribute to Voluntary Associations. I hope you will see to it that in Bristol there is a local organization ready and capable of meeting the need. The work is not easy, but it is immensely worth doing.

But this is, after all, a very limited work, and only a few of you have the time to take it up. There is a much more important task in which you can all share, and that is to create a healthy public opinion. There is still far too much of the Victorian policy of concealing mental disorder. This "hush-hush" habit is simply fatal. There are three main objectives which we want to keep before us. First of all, early treatment; next, intensive treatment; and lastly, research. Early treatment is impossible unless we can secure the confidence of the patient and the family doctor. Incalculable harm is done by delaying treatment until concealment is no longer possible. Intensive treatment means that mental hospitals must be adequately staffed and equipped with everything which medical science can devise. Bristol is fortunate, if he will allow me to say so, in its Medical Superintendent, and I hope that you will see that Dr. Barton White is given everything which he needs.

Research, to be effective, demands the co-operation of the University and the Medical School. Research in this sense means team work. It is not, as people are still apt to think, all done by an absent-minded genius breeding microbes in a test-tube. Laboratory work is essential, but it is not the whole of research. The Mental Treatment Act gives local authorities power to contribute to research, and I hope that you will see to it that this power is exercised.

The provisions of the new Act are mainly permissive, and local authorities will need the stimulus of public opinion. Bristol has in this matter a great opportunity. You have a University, a Medical School, and two great teaching hospitals. You have an abundance of clinical material for the study of mental disorder and defect of all kinds. I am very glad that a beginning has now been made at Stoke Park, and the scheme which has been undertaken there does credit to the breadth of outlook of the Administrator and Professor Berry and to the pioneer work of my old friend, the late Dr. Branthwaite.

But there is another way in which the University can help. We want more postgraduate training in psychological medicine, and there is special need of men to devote themselves to mental deficiency work. With the multiplication of colonies there will be many posts to be filled in the near future. Present students would do well to consider the possibilities which this branch of medicine offers. I do not suggest that any doctor worthy of that name takes up a particular branch of medicine merely for the sake of the money it will bring to him ; but at the same time it is idle to ignore this aspect, and I am sure that many students do not realize the opportunities of fascinating work coupled with a decent living which this branch of medical administration offers.

I have called this talk "The Modern Outlook on Mental Health." I called it that partly because I was told that it must have some title, but I think the phrase does express the spirit of what I have tried to put before you. The modern outlook on mental health is essentially hopeful. I do not mean that there is any sloppy optimism about it or an indolent waiting for something to turn up or for somebody else to do something. I mean by hope a firm, reasoned faith that the concentration of effort and of all available resources will one day solve what is one of the gravest health problems of our time. Do not look for sensational results. Years of effort, of patient labour and many disappointments will have to be faced, but for the future of our race this problem must be tackled. We cannot afford to fail, and I hope and believe that in this great task Bristol, with its University, its Medical School and great teaching hospitals, will play a worthy part.

